# The effects of diagnostic hysteroscopy on the reproductive outcomes of infertile women without intrauterine pathologies: a systematic review and meta-analysis

**DOI:** 10.4069/kjwhn.2020.12.13

**Published:** 2020-12-24

**Authors:** Soo Yeon Yang, Seung-Joo Chon, Seon Heui Lee

**Affiliations:** 1 Department of Medical Device Management and Research and Samsung Advanced Institute for Health Sciences & Technology, Sungkyunkwan University, Seoul, Korea; 2 Department of Obstetrics and Gynecology, Gil Hospital, Gachon University of Medicine, Incheon, Korea; 3 Department of Nursing Science, College of Nursing, Gachon University, Incheon, Korea

**Keywords:** Birth rate, Female infertility, Hysteroscopy, Pregnancy rate, Systematic review

## Abstract

**Purpose:**

Hysteroscopy can be used both to diagnose and to treat intrauterine pathologies. It is well known that hysteroscopy helps to improve reproductive outcomes by treating intrauterine pathologies. However, it is uncertain whether hysteroscopy is helpful in the absence of intrauterine pathologies. This study aimed to confirm whether hysteroscopy improves the reproductive outcomes of infertile women without intrauterine pathologies.

**Methods:**

We conducted a systematic review of 11 studies retrieved from Ovid-MEDLINE, Ovid-Embase, and the Cochrane Library. Two independent investigators extracted the data and used risk-of-bias tools (RoB 2.0 and ROBINS-I) to assess their quality.

**Results:**

Diagnostic hysteroscopy prior to in vitro fertilization (IVF)/intracytoplasmic sperm injection (ICSI) was associated with a higher clinical pregnancy rate (CPR) and live birth rate (LBR) than non-hysteroscopy in patients with recurrent implantation failure (RIF) (odds ratio, 1.79 and 1.46; 95% confidence interval, 1.40–2.30 and 1.08–1.97 for CPR and LBR, respectively) while hysteroscopy prior to first IVF was ineffective. The overall meta-analysis of LBR showed statistically significant findings for RIF, but a subgroup analysis showed effects only in prospective cohorts (odds ratio, 1.40 and 1.47; 95% confidence interval, 0.62–3.16 and 1.04–2.07 for randomized controlled trials and prospective cohorts, respectively). Therefore, the LBR should be interpreted carefully and further research is needed.

**Conclusion:**

Although further research is warranted, hysteroscopy may be considered as a diagnostic and treatment option for infertile women who have experienced RIF regardless of intrauterine pathologies. This finding enables nurses to educate and support infertile women with RIF prior to IVF/ICSI.

## Introduction

Infertility is defined as the failure to establish a clinical pregnancy after 12 months of regular, unprotected sexual intercourse, due to an impairment of an individual’s capacity to reproduce either alone or with his or her partner [1]. Infertility is a clinical problem that affects 13% to 15% of couples worldwide [2]. According to a recent paper describing the prevalence of infertility in 195 countries from 1990 to 2017, infertility is becoming increasingly common worldwide, rising from 1,366.85 cases per 100,000 in 1990 to 1,571.35 cases per 100,000 in 2017, a 14.962% increase [3].

Assisted reproductive technology (ART) has been developed and distributed worldwide to help infertile couples, but despite its high cost, its success rate remains low [4]. According to a report from the Centers for Disease Control and Prevention, the rate of successful embryo implantation and birth is only about 34% (43%, 35.8%, and 24.9% in patients who are 35–37, 38–40, and 41–42 years old, respectively) [5].

There are various reasons for implantation failure, including embryo quality and endometrial receptivity, but in many cases, the cause is unknown [6]. The pregnancy rate can be increased by improvements in embryo transfer and culture conditions or blastocyst selection, but these advances have not succeeded in increasing the pregnancy rate beyond 40% to 50% [7]. It is well known that intrauterine pathologies can affect the pregnancy rate in women who are using ART (in vitro fertilization [IVF] and/or intracytoplasmic sperm injection [ICSI]); therefore, it is necessary to evaluate the intrauterine environment in order to maximize the implantation rate of high-quality embryos [8].

Hysteroscopy is the gold-standard test for assessing intrauterine conditions [9]. Hysteroscopy can be used to directly and accurately diagnose abnormalities such as intrauterine adhesions, endometrial polyps, submucosal fibroids, endometritis, or uterine structural abnormalities through visualization of the cervical and intrauterine conditions, as well as through concurrent therapeutic interventions when necessary. In addition, hysteroscopy is advantageous as it can be used to perform biopsies [10].

Treating intrauterine pathologies through hysteroscopy has been found to lead to improvements in reproductive outcomes, since intrauterine lesions can negatively affect the implantation rate [11-13]. The benefits of using interventional hysteroscopy to treat intrauterine pathologies have been clearly documented in many studies [11-13]. However, no previous systematic review has determined whether hysteroscopy is helpful in improving both the clinical pregnancy rate (CPR) and the live birth rate (LBR) in the absence of intrauterine pathologies. Several systematic reviews have compared hysteroscopy and non-hysteroscopy groups [10,14-17], but none have compared diagnostic hysteroscopy with non-hysteroscopy. In 2008, a systematic review compared diagnostic hysteroscopy and non-hysteroscopy but only two randomized controlled trials (RCTs) and two non-randomized studies (NRSs) were analyzed and only the CPR was reported [18]. Even in the absence of intrauterine pathological findings, it has been hypothesized that performing hysteroscopy can help improve pregnancy rates through relaxation of the cervix, stimulation of an inflammatory reaction in the endometrium, and secretion of cytokines [19,20].

This systematic review was performed to reflect the latest results on whether diagnostic hysteroscopy prior to IVF improves the reproductive outcomes, including the LBR, of infertile women without intrauterine pathologies compared to infertile women who do not undergo hysteroscopy.

## Methods

Ethics statement: This study is a literature review of previously published studies and was therefore exempt from institutional review board approval.

### Search strategy

On January 28, 2020, a search was conducted for relevant articles regarding hysteroscopy in infertile women in the following databases: Ovid-MEDLINE, Ovid-Embase, and the Cochrane Library (the Cochrane review and trials database).

Combinations of the following Medical Subject Heading keywords were used for the searches: “hysteroscopy,” “minihysteroscopy,” “infertility,” “subfertility,” “intrauterine insemination,” “assisted conception,” “ICSI,” “fertilization in vitro or IVF,” “embryo transfer (ET),” “conception,” “miscarriage or abortion,” and “IVF-ET.”

### Inclusion and exclusion criteria

Two reviewers (SYY and SHL) independently screened the titles and abstracts of the studies extracted from the databases. The full text was subsequently reviewed to identify potential relevant articles. Studies were selected regardless of whether they reported experiences of recurrent implantation failure (RIF), and we included both RCTs and NRSs. Studies that reported the following were included: (a) infertile women who were scheduled to use ART (IVF/ICSI) for infertility treatment; (b) hysteroscopy in infertile women; and (c) the CPR or LBR in infertile women without intrauterine pathologies who underwent hysteroscopy. Additionally, only papers published within the last 20 years were included. The following types of studies were excluded: (a) animal studies; (b) articles not in English; and (c) conference posters, study protocols, review articles, cost-effectiveness analysis studies, and abstracts.

We defined the outcomes of interest before the systematic review. The primary outcome measures were the CPR and LBR, and the secondary outcome measures were the implantation and abortion rates, as well as adverse events related to hysteroscopy.

In cases of disagreement between the reviewers, discussions were held to resolve the issue. The principle was set that in cases where a consensus was not reached between the two reviewers, the third reviewer would intervene; however, all conflicts were resolved without the intervention of a third reviewer.

### Risk of bias assessment

Two reviewers (SYY and SHL) independently conducted quality assessments using the Cochrane’s risk of bias tool, ver. 2 (RoB 2.0; August 22, 2019 version) for RCTs [21]. For NRSs, the quality assessments were performed using the Cochrane’s risk of bias in non-randomized studies of interventions tool (ROBINS-I; August 1, 2016 version) [22].

The RoB 2.0 tool includes five domains; bias arising from the randomization process, bias due to deviations from the intended intervention, bias due to missing outcome data, bias due to outcome measurement, and bias due to the selection of the reported results. Each criterion for the RoB 2.0 tool was evaluated as either “low risk,” “high risk,” or “some concerns.” The ROBINS-I tool includes seven domains; bias due to confounding, bias due to the selection of the participants, bias in the classification of the interventions, bias due to deviations from the intended interventions, bias due to missing data, bias in measurement of outcomes, and bias in selection of the reported result. Each item was graded as “low risk,” “moderate risk,” “serious risk,” “critical risk,” or “no information.” Disagreements regarding the quality assessments between the reviewers were resolved through discussion.

### Data extraction and statistical analysis

Two reviewers (SYY and SHL) independently extracted data from the studies selected according to the selection criteria. Disagreements between the reviewers were resolved through discussions. The following data were extracted for each of the 11 selected studies: author; year of publication; title; country in which the study was conducted; study design, and group; number and ages of the patients; experiences of RIF; previous investigations (diagnostic tests performed before participation in the study such as transvaginal ultrasonography [TVS] or hysterosalpingography [HSG]); descriptions of the participants (inclusion and exclusion criteria, type of infertility); details of the intervention (hysteroscopy or no hysteroscopy); whether endometrial stimulation was performed; the method used to attempt pregnancy; the author’s conclusion; the main outcome measures; intergroup differences; and adverse events of hysteroscopy.

The authors of the selected studies were contacted to provide missing or unclear information on the trial methods or data. We used the meta-analyses of observational studies in epidemiology reporting guidelines [23].

The pooled odds ratio (OR) was extracted for categorical data. Meta-analysis was undertaken where there were two or more studies. From each study, binary data were extracted in 2×2 tables and the results were pooled and expressed as ORs with 95% confidence intervals (CIs) using a random-effects model, as appropriate [24]. Heterogeneity analyses were performed using forest plots, and the I^2^ statistic was used to quantify the heterogeneity between studies [25]. All statistical analyses were performed using RevMan ver. 5.4 software (Cochrane, London, UK).

## Results

### Study characteristics

**Figure 1.**
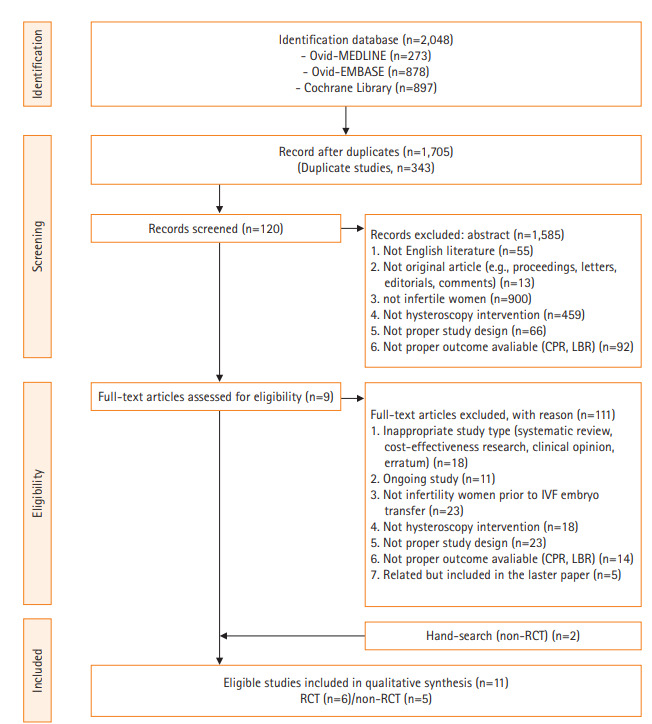
Preferred Reporting Items for Systematic Reviews and Meta-Analyses (PRISMA) flow diagram. CPR: Clinical pregnancy rate; ICSI: intracytoplasmic sperm injection; IVF: in vitro fertilization; LBR: live birth rate; NRS: non-randomized study; RCT: randomized controlled trial.

The process of study selection is summarized in Figure 1.

A total of 2,048 studies were initially identified. After excluding duplicates, 1,705 studies remained. A total of 120 studies were selected upon initial screening. After the full-text review, 111 studies were excluded and nine studies were included, with two studies additionally included based on a hand search (March 10, 2020). Ultimately, a total of 11 studies were included [26-36]. The basic characteristics of the included studies are shown in Supplementary Table 1.

Six RCTs [26,27,29,32,34,35] and five NRSs [28,30,31,33,36], respectively, were selected that investigated the CPR or LBR in infertile women without intrauterine lesions after hysteroscopy. Of the 11 studies that were included, four (36.4%) were conducted in Turkey [26,30,33,36] and two (18.2%) in Iran [31,35], and one each was conducted in Egypt [29], Greece [28], India [27], the Netherlands [34], and Europe [32]. Six studies (54.5%) included infertile women who had experienced RIF [26-28,31-33], and three (27.3%) included infertile patients who were undergoing IVF for the first time [34-36]. Two studies (18.2%) did not separately define whether the patients had experienced RIF or were undergoing IVF for the first time [29,30]. IVF/ICSI was performed after hysteroscopy in all of the studies that performed a normal TVS or HSG assessment of the uterine cavity. The purpose of our study was not to compare interventional hysteroscopy and non-hysteroscopy to treat abnormal pathologies such as polyps and adhesions, so we did not investigate abnormal findings separately.

### Characteristics of the intervention

Of the 11 studies included in our systematic review, two (18.2%) performed endometrial stimulation during hysteroscopy [27,29]. In one of the two studies, sampling of the endometrium by aspiration using a 4-mm cannula was performed at the end of the procedure, and the samples were sent for histological evaluation [27]. In the other study, the endometrial biopsy was performed using biopsy forceps under direct visualization [29].

In the hysteroscopy intervention group, ART (IVF/ICSI) was performed after hysteroscopy in the initial proliferative phase. In the non-hysteroscopy group, the attempt to use ART was made immediately in 10 studies, with the exception of one study [28].

Regarding embryo transplantation, fresh embryos were transplanted in nine studies [26,27,29,30,32-36] and fresh or frozen embryos were transplanted in two studies [28,31].

A 2.9- to 5.5-mm-diameter hysteroscope was used in the intervention group. Four and three studies (36.4% and 27.3%, respectively) used a 4-mm and 5-mm-diameter hysteroscope, respectively [26,27,30,31,34,35]. One study (9.1%) did not mention the diameter of the hysteroscope used [36]. The characteristics of the intervention are summarized in Table 1.

### Result of risk of bias assessment

Upon quality assessment, three of the six RCTs [26,27,35] were graded as having “some concerns” for selection bias (bias arising from the randomization process) because the allocation concealment information could not be confirmed, but the imbalances at baseline did not suggest any problems. The other three studies [29,32,34] were graded as “low risk” for selection bias. In all six RCTs [26,27,29,32, 34,35], performance bias (bias due to deviations from the intended intervention) and detection bias (bias in measurement of the outcome) were both graded as “low risk.” In the evaluation of attrition bias (bias due to missing outcome data), one [29] of the six studies were evaluated as having “some concern” because an intention-to-treat analysis was not conducted, and five studies [26,27,32,34,35] were evaluated as “low risk.” Two studies [32,34] were rated as “low risk” for reporting bias (bias in selection of the reported result), while four studies [26,27,29,35] were rated as having “some concern” because they did not report selected results, and there was no information as to whether the analysis was performed according to a predefined plan.

Of the five NRSs, four [28,31,33,36] were classified as “moderate risk” for bias due to confounding (the preintervention domain in confounding) because the confounding variables were not properly measured and controlled, although the measurement of the important domains was sufficiently reliable and valid. In one study [30], even though IVF was performed, the confounding variables for whether the patients experienced RIF were not identified; therefore, it was graded as having “serious risk.” Biases due to deviations from the intended interventions (the postintervention domain in confounding) were graded as “low risk” in all five studies [28,30,31,33,36]. For bias in selection of participants into the study (the preintervention domain of selection bias), three studies [28,33,36] were rated as “moderate risk”. One [28] out of these three studies had moderate risk because the selection of the patients for the study may have been related to the intervention (hysteroscopy) and it was not possible to determine whether adjustment techniques were used to correct for the presence of selection bias. The remaining two [33,36] were determined to have moderate risk although they applied the inclusion/exclusion criteria regardless of the interventions or outcomes; however, as they were retrospective studies, the start of the follow-up period and intervention did not coincide. Two studies [30,31] were evaluated as “low risk.” Biases due to missing data (the postintervention domain in selection bias) were graded as “low risk” in all five studies. Two studies [30,31] were at a low risk for bias in the classification of the interventions (the intervention domain in information bias). Three studies [28,33,36] were graded as having “moderate risk” because although the intervention status was well defined, some aspects regarding the assignment of the intervention status were determined retrospectively. Bias in the measurement of outcomes (the postintervention domain for information bias) was graded as “low risk” in all five studies because the outcome measures e.g., the CPR and LBR involved negligible assessor judgment. As for the bias in the selection of the reported results (reporting bias), four studies [30,31,33,36] were evaluated as “moderate risk” because their pre-registered protocol or statistical analysis plans could not be identified. In one study [28], even though the study period was long enough (6 years), the LBR was not reported, and this was graded as a “serious risk.” The results of the quality assessment are presented in detail in Supplementary Figure 1.

### Primary outcome measures: CPR and LBR

#### Diagnostic hysteroscopy vs. non-hysteroscopy according to the number of IVF attempts

Diagnostic hysteroscopy vs. non-hysteroscopy was analyzed by subgroup according to IVF attempts.

##### 1) CPR

The same seven studies (four RCTs [26,27,29,35] and three NRSs [28,31,36]) of 1,549 (out of a total of 3,152) infertile women reported the CPR in women who underwent diagnostic hysteroscopy and were included in the analysis. The remaining 1,603 were the non-hysteroscopy group.

**Figure 2.**
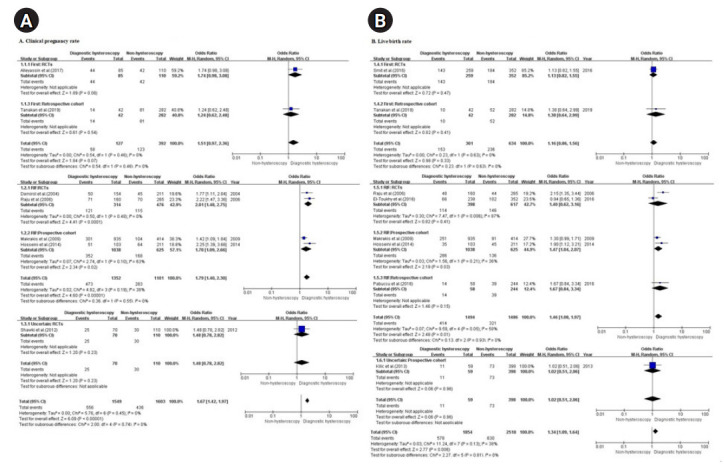
Diagnostic hysteroscopy vs. non-hysteroscopy according to the number of in vitro fertilization attempts. (A) Clinical pregnancy rate. (B) Live birth rate. df: Degree of freedom; M-H: Mantel-Haenszel; RCT: randomized controlled trial; RIF: recurrent implantation failure.

The overall meta-analysis of the seven studies showed that the RIF group [26-28,31] had a significant difference in the CPR, while the group of women undergoing their first IVF attempts [35,36] did not (OR, 1.79 and 1.51; 95% CI, 1.40–2.30 and 0.97–2.36 for RIF and first attempts, respectively). A subgroup analysis of the RIF group showed effectiveness in both RCTs [26,27] and prospective cohorts [28,31] (OR, 2.01 and 1.70; 95% CI, 1.48–2.75 and 1.09–2.66 for RCTs and prospective cohorts, respectively) while a subgroup analysis of the first-attempt group showed ineffectiveness in both an RCT [35] and a retrospective cohort [36] (OR, 1.74 and 1.24; 95% CI, 0.98–3.08 and 0.62–2.48 for the RCT and retrospective cohort, respectively) (Figure 2-A).

##### 2) LBR

Eight of the 11 studies (three RCTs [27,32,34] and five NRSs [28,30,31,33,36]) reported the LBR in women who underwent diagnostic hysteroscopy and were included in the analysis. In total, 4,372 infertile women were included in the eight studies: 1,854 in the diagnostic hysteroscopy group without intrauterine pathologies and 2,518 in the non-hysteroscopy group.

The overall meta-analysis of the eight studies showed that the RIF group [27,28,31-33] had a significant difference in the LBR, while the first-attempt group [34,36] did not (OR, 1.46 and 1.16; 95% CI, 1.08–1.97 and 0.86–1.56 for RIF and first attempts, respectively). A subgroup analysis of RIF group showed effectiveness in prospective cohorts [28,31], but not in RCTs [27,32] or a retrospective cohort [33] (OR, 1.47, 1.40, and 1.67; 95% CI, 1.04–2.07, 0.62–3.16, and 0.84–3.34 for prospective cohorts, RCTs, and the retrospective cohort, respectively). A subgroup analysis of the first-attempt group showed ineffectiveness in both an RCT [34] and a retrospective cohort [36] (OR, 1.13 and 1.38; 95% CI, 0.82–1.55 and 0.64–2.99 for the RCT and retrospective cohort, respectively) (Figure 2-B.).

#### Diagnostic hysteroscopy vs. non-hysteroscopy in women who underwent endometrial stimulation during hysteroscopy

Diagnostic hysteroscopy vs. non-hysteroscopy was analyzed by subgroup according to whether endometrial stimulation was performed during hysteroscopy.

##### 1) CPR

Seven of the 11 studies (four RCTs [26,27,29,35] and three NRSs [28,31,36]) reported the CPR in patients who underwent diagnostic hysteroscopy and were included in the analysis. In total, 3,152 infertile women were included in the seven studies: 1,549 in the diagnostic hysteroscopy group without intrauterine pathologies and 1,603 in the non-hysteroscopy group.

**Figure 3.**
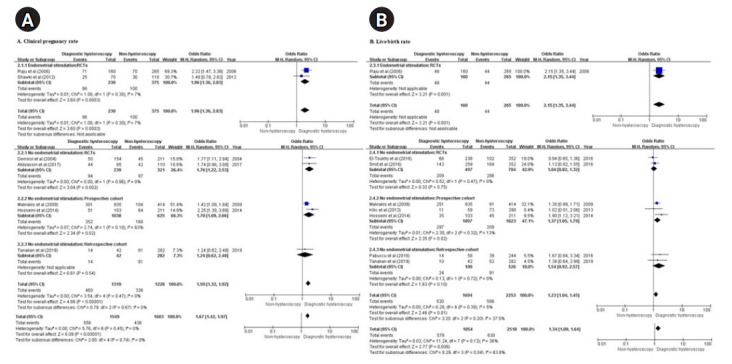
Diagnostic hysteroscopy vs. non-hysteroscopy in patients who underwent endometrial stimulation during hysteroscopy. (A) Clinical pregnancy rate. (B) Live birth rate. df: Degree of freedom; M-H: Mantel-Haenszel; RCT: randomized controlled trial.

The results of the seven studies showed significant differences in the CPR regardless of whether endometrial stimulation was performed in the diagnostic hysteroscopy group without intrauterine pathologies before IVF/ICSI when compared with the non-hysteroscopy group (OR, 1.67, 95% CI, 1.42-1.97; I^2^=0%, *p*=.45). The degree of improvement in the CPR observed after endometrial stimulation during hysteroscopy [27,29] seemed to be higher than that observed after no endometrial stimulation during hysteroscopy [26,28,31,35,36] (OR, 1.96 and 1.59; 95% CI, 1.36–2.83 and 1.32–1.92 for endometrial stimulation and no endometrial stimulation, respectively). The subgroup analysis of RCTs showed effectiveness for the CPR regardless of endometrial stimulation (OR, 1.96 and 1.76; 95% CI, 1.36–2.83 and 1.22–2.53 for endometrial stimulation [27,29] and no endometrial stimulation [26,35], respectively) (Figure 3-A).

##### 2) LBR

As reported above, the same eight studies (three RCTs [27,32,34] and five NRSs [28,30,31,33,36]) reported the LBR after diagnostic hysteroscopy without intrauterine pathology (1,854 out of a total of 4,272 infertile women) and were included in the analysis. The remaining 2,518 were the non-hysteroscopy group.

The results of the eight studies showed significant differences in the LBR regardless of endometrial stimulation in the hysteroscopy group without intrauterine pathologies before IVF/ICSI when compared with the non-hysteroscopy group, but the degree of significance was not as high as it was for the CPR (OR, 1.34; 95% CI, 1.09–1.64; I^2^=38%, *p=*.13). The degree of improvement in the LBR observed after endometrial stimulation during hysteroscopy [27] seemed to be higher than that observed after no endometrial stimulation during hysteroscopy [28,30-34,36] (OR, 2.15 and 1.23; 95% CI, 1.35–3.44 and 1.04–1.45 for endometrial stimulation and no endometrial stimulation, respectively). A subgroup analysis of the patients who did not undergo endometrial stimulation showed ineffectiveness in RCTs [32,34] and retrospective cohorts [33,36], but effectiveness in prospective cohorts [28,30,31] (OR, 1.04, 1.54, and 1.37; 95% CI, 0.82–1.32, 0.92–2.57, and 1.05–1.79 for RCTs, retrospective cohorts, and prospective cohorts, respectively) (Figure 3-B).

### Secondary outcome measures: implantation rate, miscarriage rate, and adverse events

#### Implantation rate

The implantation rate was reported for the hysteroscopy groups, but no study separately reported the implantation rate of infertile patients without intrauterine pathologies (diagnostic hysteroscopy), so this parameter was excluded from the analysis.

#### Diagnostic hysteroscopy vs. non-hysteroscopy: miscarriage rate

Three of the 11 studies (2 RCTs, 1 NRSs [26,27,31]) reported the miscarriage rate in patients who underwent diagnostic hysteroscopy and were included in the analysis. In total, 820 infertile women were included in these three studies; 328 in the hysteroscopy group without intrauterine pathologies and 492 in the control group.

**Figure 4.**
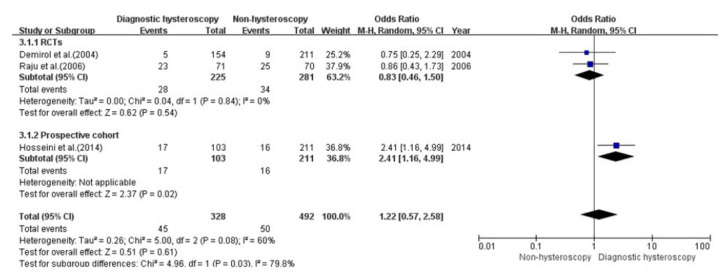
Meta-analysis of the miscarriage rate. df: Degree of freedom; M-H: Mantel-Haenszel; RCT: randomized controlled trial.

A subgroup analysis was performed with RCTs and NRSs, as high heterogeneity was found (*p=*.08, I^2^=60%). The results of the meta-analysis of the miscarriage rate are shown in Figure 4.

The results of the three studies did not show a significant difference in the miscarriage rate in the diagnostic hysteroscopy group without intrauterine pathologies compared with the non-hysteroscopy group (OR, 1.22; 95% CI, 0.57–2.58; I^2^=60%, *p=*.08).

#### Adverse events relating to hysteroscopy

Seven studies (63.6%) did not mention any adverse events relating to hysteroscopy [28-31,33,35,36]. Of remaining four studies that noted adverse events in the hysteroscopy group, there were no adverse events in two studies [27,32], while two other studies (18.2%) reported that patients developed pain [26] and endometritis (n=1, <1%) [34]. No studies, however, separately reported the adverse events of infertile patients without intrauterine pathologies, so this pa­rameter was excluded from the analysis.

## Discussion

This study is the first systematic review and meta-analysis to compare the reproductive outcomes of infertile patients without intrauterine pathologies who underwent hysteroscopy (diagnostic hysteroscopy) and groups of infertile patients who did not undergo hysteroscopy (non-hysteroscopy) since the systematic review conducted by El-Toukhy et al. [18] in 2008. El-Toukhy et al. [18] reported only the CPR and included two RCTs and two NRSs due to the limitation of the number of related studies at the time of the systematic review, and it was not possible to conduct an analysis according to the number of IVF attempts. This systematic review included the results of nine recent studies (four RCTs, five NRSs) including two RCTs with a low risk of bias [32,34] since 2008 and both the CPRs and the LBRs were analyzed. Other previous systematic reviews have compared groups of patients who did or did not receive hysteroscopy (hysteroscopy vs. non-hysteroscopy) [10,14-17]. In the previous systematic reviews, the results of interventional hysteroscopy to treat intrauterine abnormalities and diagnostic hysteroscopy in patients without intrauterine pathologies were combined and compared with the non-hysteroscopy group [10,14-17]. Di Spiezio Sardo et al. [14] compared diagnostic hysteroscopy and interventional hysteroscopy and found a higher pregnancy rate in the interventional hysteroscopy group in which intrauterine pathologies were removed. However, that previous study did not analyze whether hysteroscopy is helpful even in the absence of intrauterine pathologies compared with the non-hysteroscopy group.

This study showed that performing diagnostic hysteroscopy prior to IVF/ICSI may improve the CPR and LBR even in patients without intrauterine pathologies, as opposed to not performing hysteroscopy, especially in patients with RIF; however, hysteroscopy prior to the first IVF attempt was found to be ineffective. A subgroup analysis was conducted according to whether endometrial stimulation was performed during hysteroscopy to determine whether endometrial biopsy affects reproductive outcomes when diagnostic hysteroscopy is performed in infertile women without intrauterine pathologies. Regardless of endometrial stimulation, the hysteroscopy group showed greater improvement in the CPR and LBR than the non-hysteroscopy group.

### The impact of the number of IVF attempts

Regarding the number of IVF attempts, our study showed that the CPR after diagnostic hysteroscopy was effective in patients who had experienced RIF without intrauterine pathologies (in comparison to no hysteroscopy), but not in infertile women without intrauterine pathologies attempting IVF for the first time (OR, 1.79 and 1.51; 95% CI, 1.40–2.30 and 0.97–2.36 for RIF and first attempts, respectively). The CPR was assessed in seven studies with 3,152 participants. Our findings are supported by recent systematic reviews by Cao et al. [15] and Mao et al. [17] reporting that hysteroscopy in infertile women experiencing RIF improved CPR compared to non-hysteroscopy groups. Pundir et al. [10] reported that the CPR was higher in infertile women who underwent hysteroscopy prior to the first IVF attempt than in the non-hysteroscopy group. However, their meta-analysis was conducted with four NRSs and one RCT, which was a conference abstract, due to the limitation of the number of studies at the time of the systematic review in 2014. Pundir et al. [10] also mentioned that the degree of improvement was lower in patients attempting IVF for the first time than in those with previous IVF failure, in accordance with the systematic review of El-Toukhy et al. [37]. Thus, a high-quality randomized trial is necessary.

This study also showed that performing diagnostic hysteroscopy prior to IVF/ICSI for women with RIF may improve the LBR even in the absence of intrauterine pathologies compared with the non-hysteroscopy group, whereas hysteroscopy prior to the first IVF attempt was found to be ineffective (OR, 1.46 and 1.16; 95% CI, 1.08–1.97 and 0.86–1.56 for RIF and first attempts, respectively). However, the subgroup analysis showed effectiveness only in prospective cohorts (OR, 1.40 and 1.47; 95% CI, 0.86–1.56 and 1.04–2.07 for RCTs and prospective cohorts, respectively). The LBR was also assessed in eight studies with 4,372 participants. Regarding the effects on the LBR in women with RIF, the results of previous systematic reviews are discordant. Cao et al. [15] analyzed RCT and prospective cohorts together and showed an effect in the RIF group, which is consistent with our study, although they did not separately analyze only the diagnostic hysteroscopy group without intrauterine pathologies compared to the non-hysteroscopy group. Systematic reviews that analyzed only RCTs were conducted by several studies: Di Spiezio Sardo et al. [14] and Kamath et al. [16] showed improvements in the LBR in the RIF group (diagnostic hysteroscopy). Saleh et al. [38] showed no improvement in the LBR in the RIF group, but included only two RCTs [27,32], whereas Kamath et al. [16] showed an effect; however, they included the results reported by Aghahosseini et al. [39] as well as two RCTs [27,32]. The study of Aghahosseini et al. [39] was excluded in this systematic review, as it is a conference abstract.

Di Spiezio Sardo et al. [14] and Kamath et al. [16] reported an effect on the CPR in first IVF attempts group, but not in the LBR. However, the systematic review conducted by Di Spiezio Sardo et al. [14] in 2016 did not include two RCTs from that same year [32,34], and Kamath et al. [16] reported that screening hysteroscopy may benefit women with two or more IVF failures in a subgroup analysis.

Studies classified as having some concerns in RoB 2.0 [26,27] showed that diagnostic hysteroscopy prior to IVF may be beneficial for the CPR in the RIF group, but not for women attempting IVF for the first time. Studies assessed as having serious [28] and moderate [31] risk in ROBINS-I showed that diagnostic hysteroscopy prior to IVF may be beneficial for the LBR in the RIF group, but not in the first-time IVF group. Our findings should be interpreted with caution, and verification of the effectiveness of diagnostic hysteroscopy in a larger multicenter randomized clinical study in the future is recommended. El-Toukhy et al. [18] noted in their 2008 systematic review that the benefit of hysteroscopy before IVF was lower in infertile patients undergoing IVF for the first time than in infertile patients who had experienced RIF. It has been pointed out that a higher number of IVF failures is indicative of an increased risk of intrauterine pathology, which may be related to the ability of hysteroscopy to reliably detect and potentially treat intrauterine pathologies. In this study, the same result was obtained even though hysteroscopy was not used to correct intrauterine pathologies. Therefore, we suspect that other factors may affect the endometrial receptivity of infertile patients who have experienced RIF that are absent in women undergoing IVF for the first time. Further research is needed on the factors that specifically affect endometrial receptivity in infertile women who have experienced RIF, as distinct from women undergoing IVF for the first time.

### Impact of endometrial stimulation during diagnostic hysteroscopy

With regard to endometrial stimulation during hysteroscopy, this study showed improvements in the CPR and LBR regardless of endometrial stimulation (OR, 1.67 and 1.34, 95% CI, 1.42-1.97 and 1.09–1.64 for CPR and LBR, respectively). This result is consistent with the systematic review of Kamath et al. [16], although they did not separately analyze only the diagnostic hysteroscopy group without intrauterine pathologies compared to the non-hysteroscopy group. El-Toukhy et al. [18] explained that the fertility-enhancing effect of hysteroscopy could also be independent of whether intrauterine pathologies are corrected and might be related to a number of other factors. One of several hypotheses is that injury during hysteroscopy may trigger the massive secretion of growth factors and cytokines, which may be beneficial for embryo implantation [20,40]. Mechanical endometrial injury may enhance endometrial receptivity by modulating the expression of gene encoding factors required for implantation, such as glycodelin A, laminin alpha-4, integrin alpha-6, and matrix metalloproteinase-I [41,42]. One study reported that when endometrial biopsies were performed repeatedly, Cx43 (a gap junction protein that could be a possible parameter for successful implantation and may predict implantation competence) was expressed; which could help improve the reproductive outcomes and pregnancy rates [43]. Shohayeb et al. [44] did not separately report outcomes for infertile women without intrauterine pathologies but showed a significantly higher implantation rate, CPR, and LBR after endometrial stimulation during hysteroscopy prior to ICSI (single endometrial biopsy regimen) for infertile women in comparison to hysteroscopy without endometrial scraping. Various mechanisms have been proposed to support the hypothesis that endometrial scratch injuries may improve endometrial receptivity. The most recent hypothesis is the backward development hypothesis, according to which an endometrial scratch injury may delay endometrial maturation, minimizing the negative effects of ovarian stimulation and implantation [45-47]. Another hypothesis based on animal models posits that injury may induce the rapid growth of endometrial cells in a similar fashion to that of decidual cells in humans [48,49].

A subgroup analysis was performed according to whether endometrial stimulation during hysteroscopy. The degree of improvement in IVF outcomes observed after endometrial stimulation during hysteroscopy seemed to be higher than that after no endometrial stimulation during hysteroscopy (OR, 1.96 and 1.59; 95% CI, 1.36–2.83 and 1.32–1.92 for the CPRs after endometrial stimulation and no endometrial stimulation, respectively; OR, 2.15 and 1.23; 95% CI, 1.35–3.44 and 1.04–1.45 for the LBRs after endometrial stimulation and no endometrial stimulation, respectively). The CPR was assessed in seven studies with 3,152 participants and the LBR was also assessed in eight studies with 4,372 participants, but only two RCTs investigated endometrial stimulation during hysteroscopy [27,29], and only one RCT reported the LBR [27]. Due to the limitation that only one study with endometrial stimulation reported the LBR [27], it cannot be said that endometrial stimulation during hysteroscopy has an additional benefit on the LBR compared to no scratching during hysteroscopy. However, given the hypothesis that endometrial scratch injuries may have beneficial effects, it is necessary to confirm the effects of endometrial stimulation during hysteroscopy through a large-scale randomized study in the future.

Our study showed that diagnostic hysteroscopy alone prior to IVF may improve reproductive outcomes even in the absence of intrauterine pathologies, compared with patients who did not undergo hysteroscopy. In addition to the hypothesis of cytokine and growth factor release due to the injury induced by hysteroscopy, three hypotheses have been proposed to explain the improvement of reproductive outcomes resulting from diagnostic hysteroscopy even if an intrauterine pathology is not corrected. First, the saline used during hysteroscopy mechanically removes the harmful anti-adhesive glycoprotein molecules involved in endometrial receptivity from the endometrial surface (cyclooxygenase-2, mucin-I, and integrin αVβ3) [50]. Thus, the effect of saline irrigation may lead to improved endometrial conditions and mechanical stimulation of the endometrium, which may enhance endometrial receptivity beyond correcting intrauterine pathologies [50]. Of the 11 studies included in this systematic review, nine (81.8%) reported that normal saline was used as the distension media [26,28-35], one study indicated that glycine was used [27], and another study only stated that diagnostic hysteroscopy was performed [36]. The CPR and LBR were significantly higher than in the non-hysteroscopy group when hysteroscopy prior to IVF was performed in infertile women without intrauterine pathologies (OR, 1.67 and 1.09; 95% CI, 1.42–1.97 and 1.09–1.64 for CPR and LBR, respectively). The second hypothesis is that benefits may occur because hysteroscopy allows more accurate embryo placement and easier embryo transfer by confirming the shape of the uterus and measurement of uterine cavity length [51]. The final hypothesis notes that introducing the hysteroscope through the cervical canal into the uterine cavity could facilitate future embryo transfer, which is the final and most crucial step in IVF [51]. Cervical canal dilatation has been shown to reduce difficulties in embryo transfer, thus increasing the likelihood of pregnancy after IVF [19]. To determine whether reproductive outcomes are improved by cervical dilatation, future RCTs should compare hysteroscopy and cervical dilatation only.

### Limitations

Despite our findings, this study has several limitations. First, some studies did not separately investigate infertile women with intrauterine pathologies after hysteroscopy regarding the CPR, LBR, implantation, and miscarriage rates separately; therefore, not all of the data were limited to infertile women without intrauterine pathologies who underwent hysteroscopy before ART compared with the non-hysteroscopy group. We tried to contact authors to obtain this information, but no response was received. Nonetheless, this study is meaningful as it is the first systematic review to quantify the effect of hysteroscopy on both the CPR and LBR in infertile women without intrauterine pathologies. Second, heterogeneity was shown when pooling results for the LBR from the eight included studies (*p=*.13, I^2^= 38%). These eight studies included three RCTs (two with a low risk of bias and one with some concerns) and five NRSs. A sensitivity analysis was performed to control for the impact of confounding variables by omitting a study [27], and heterogeneity was eliminated upon its exclusion (*p=*.39, I^2^=5%). The difference between that study and the other studies was that endometrial biopsy was performed during hysteroscopy. Therefore, a subanalysis was performed according to whether endometrial stimulation was performed during hysteroscopy. The degree of improvement in IVF outcome observed after endometrial stimulation during hysteroscopy seemed to be higher than that observed after no endometrial stimulation during hysteroscopy; however, the evidence for this difference is low-quality, and future studies should confirm the effect of endometrial biopsy during hysteroscopy.

In conclusion, this systematic review and meta-analysis showed that performing diagnostic hysteroscopy prior to IVF/ICSI may improve the CPR and LBR as opposed to not performing hysteroscopy, even in the absence of intrauterine pathologies, especially in patients with RIF; however, hysteroscopy prior to the first IVF attempt was found to be ineffective. In addition, stimulation of the endometrium during hysteroscopy may improve reproductive outcomes. However, large-scale randomized studies are needed to provide stronger evidence in the future. Although further research is needed, hysteroscopy may be considered as a diagnostic and treatment option for infertile women who have experienced RIF regardless of the presence of intrauterine pathologies, and endometrial biopsy could be considered when performing hysteroscopy. Hysteroscopy has few adverse events, as confirmed in this systematic review, but infertile women may feel fear and anxiety before hysteroscopy and might doubt whether hysteroscopy can improve reproductive outcomes. If infertile women who have experienced RIF are scheduled for hysteroscopy before IVF/ICSI, nurses can not only provide emotional support by telling patients that adverse effects of hysteroscopy are rare, inform them that hysteroscopy may have a beneficial effect on reproductive outcomes even if there is no intrauterine pathology to be treated, may also alleviate their fears.

## Figures and Tables

**Table 1.** The characteristics and effectiveness of the reviewed interventions (N=11)

First author (year)	Intervention		Comparator	Endometrial irritation (I only)	Method of pregnancy attempt (both I and C)	Embryo / day of ET	Authors’ conclusion	Main outcome measures	Intergroup differences	Adverse events of hysteroscopy
	Timing	Hysteroscope								
Tanacan (2019) [36]	In the early to midfollicular phase of the menstrual cycle (1–3 months before the start of IVF)	Not specified	Without diagnostic hysteroscopy prior to the first IVF cycle	No scratching	IVF	Fresh embryo / day 3 or day 5	OH before the first IVF treatment cycle did not improve fertility outcomes in patients without previously detected pathology of the uterine cavity.	(1) Implantation rate	(1) .840	Not specified
		Distension medium: not specified					Routine usage of hysteroscopy should not be offered to patients in their first IVF cycles.	(2) CPR	(2) .541	
								(3) LBR	(3) .420	
Alleyassin (2017) [35]	Between the 18th and 22nd day of their menstrual cycles (mid-luteal phase) before ICSI cycles	4-mm diameter diagnostic sheath, continuous flow, rigid, 30° view (Karl Storz, Tuttlingen, Germany)	Did not undergo OH before ICSI cycles	No scratching	ICSI	Fresh embryo / day 3	Routine OH before ICSI cycles provided direct evaluation of uterine cavity.	(1) CPR	(1) .004	Not specified
		Distension medium: Saline					CPR improved after correction of endometrial cavity abnormalities.	(2) Miscarriage rate	(2) NS	
El-Toukhy (2016) [32]	Before controlled ovarian stimulation for IVF	2.9-mm diameter, rigid 30° view, with an atraumatic tip (TROPHY scope; Karl Storz)	Immediate controlled ovarian stimulation for IVF/ICSI	No scratching	IVF (with or without ICSI)	Fresh embryo /	Routine OH did not improve IVF outcomes in women with RIF who had a normal uterine ultrasound scan.	(1) Pregnancy rate	(1) .86	No hysteroscopy-related adverse events
	Within 14 days of menstruation	Distension medium: saline				When it is considered top quality (day 2 or days 3–4 or days 5–6)		(2) CPR	(2) .65	
								(3) LBR (after 1 cycle of IVF)	(3) .96	
Smit (2016) [34]	In the early-mid follicular phase of a menstrual cycle (days 3–12)	5-mm outer diameter continuous flow hysteroscope with a 5-Fr working channel and a 30° direction of view	Immediate start of IVF	No scratching	IVF	Fresh embryo / not specified	Routine OH before the first IVF or ICSI treatment cycle did not improve fertility prospects in infertile women with a normal TVS of the uterine cavity who had not had a previous hysteroscopy.	(1) Implantation rate	(1) .23	One (<1%) woman: endometritis after hysteroscopy
	1–3 months before the start of IVF treatment	Distension medium: saline						(2) CPR	(2) .71	
								(3) OPR	(3) .69	
								(4) LBR	(4) .75	
Pabuçcu (2016) [33]	In early follicular phase (1–6 months before the beginning of a new cycle)	4-mm outer diameter, rigid, continuous flow; 30° forward and oblique view	Immediately started a new ART cycle	No scratching	IVF/ICSI	Fresh embryo / day 3 or day 5	Unrecognized intrauterine pathologies can be easily detected and concurrently treated during the OH procedure with high success rates.	(1) Implantation rate	(1) .38	Not specified
		Distension medium: saline					The overall beneficial impact in terms of reproductive outcomes seems to depend on the extent of the pathology.	(2) Chemical pregnancy rate	(2) .08	
								(3) LBR	(3) .06	
								(4) Miscarriage rate	(4) .26	
Hosseini (2014) [31]	In the menstrual cycle just before ovarian stimulation or endometrial preparation	4-mm rigid, continuous flow, 30° forward, and oblique view	Hysteroscopy was not performed	No scratching	ART IVF/ET	Fresh or frozen embryo / day 3	OH before fresh cycles and frozen-thawed cycles in women experiencing RIF with apparently normal uterine cavity significantly increased the pregnancy rates.	(1) Chemical pregnancy rate	(1) <.001	Not specified
		Distension medium: saline						(2) CPR	(2) .001	
								(3) Delivery rate	(3) .026	
Kilic (2013) [30]	Assessed prior to IVF	4-mm (Karl Storz)	Underwent IVF without OH evaluation	No scratching	IVF	Not specified	OH before IVF can detect and treat intrauterine pathologies, with positives effect on pregnancy outcomes.	(1) LBR	(1) <.05	Not specified
	Follicular phase (days 5–7 of menstrual cycle)	Distension medium: saline								
Shawki (2012) [29]	The early postmenstrual period before controlled ovarian stimulation for ICSI	3.5 mm with a 0° grade (Versascope; Gynecare, Ethicon, Sommerville, NJ, USA)	Immediate controlled ovarian stimulation for ICSI	Endometrial biopsy	ICSI	Fresh embryo / not specified	Improvement in implantation and CPR were observed after OH prior to ICSI.	(1) CPR	(1) <.05	Not specified
		Optic Illumination (250-W Xenon light source)					Routine OH should be an essential step of the infertility workup before ART even in patients with normal HSG and/or TVS.	(2) Implantation rate	(2) <.05	
		Distension medium: saline								
Makrakis (2009) [28]	Less than 12 months before the first IVF attempts	2.9-mm, 30° angle, external sheath of 5.5-mm diameter providing inflow and outflow (Karl Storz)	Matched control (no hysteroscopy before IVF cycles)	No scratching	IVF	Fresh or frozen embryo / day 3–5	Hysteroscopy could be seen as a positive prognostic factor for achieving a subsequent IVF pregnancy in women with a history of two consecutive implantation failures.	(1) CPR	(1) .04	Not specified
	Shortly after cessation of menses	Distension medium: saline						(2) OPR	(2) .06	
Rama Raju (2006) [27]	The early proliferative phase before controlled ovarian stimulation for IVF treatment	5-mm diameter, 1.9-mm miniature, 30° view, 3 mm Bettocchi continuous flow sheath with an incorporated 5-Fr working channel (Karl Storz)	Immediate controlled ovarian stimulation for IVF treatment	Endometrial biopsy	IVF	Fresh embryo / day 3	Patients with recurrent IVF-ET failures after normal HSG should also be reevaluated using hysteroscopy prior to commencing IVF-ET cycles in order to enhance the CPR.	(1) CPR	(1) <.05	No further complications
		Distension medium: glycine						(2) Miscarriage rate	(2) NS	
								(3) LBR	(3) <.05	
Demirol and Gurgan (2004) [26]	The early proliferative phase before controlled ovarian stimulation for IVF treatment	5-mm continuous flow, lens diameter 2.9-mm, 30° view, 5-mm diameter sheath, Bettocchi, size 5 (Karl Storz)	Immediate controlled ovarian stimulation for IVF treatment	No scratching	IVF	Fresh embryo / day 3	Patients with normal HSG but recurrent IVF-ET failure should be evaluated prior to commencing IVF-ET cycles to improve the clinical PR.	(1) Number of clinical pregnancies	(1) <.05	Mild pain resembling menstrual cramps
	(2–6 months after the last failed IVF cycles)	Distension medium: saline						(2) Number of first trimester abortions	(2) NS	

ART: Artificial reproductive technology; C: control; CPR: clinical pregnancy rate; ET: embryo transfer; Fr: French guage; HSG: hysterosalpingography; I: intervention; ICSI: intracytoplasmic sperm injection; IVF: in vitro fertilization; LBR: live birth rate; NS: not significant; OH: office hysteroscopy; OPR: ongoing pregnancy rate; PR: pregnancy rate; RIF: recurrent implantation failure; TVS: transvaginal sonography.

## Supplementary materials

Further details on supplementary materials are presented online (available at https://doi.org/10.4069/kjwhn.2020.12.13).

Supplementary Table 1. Characteristics of the included studies (N=11)

Supplementary Figure 1. Risk of bias. (A) Randomized controlled studies; summary. (B) Randomized controlled studies; by study. (C) Non-randomized studies; summary. (D) Non-randomized studies; by study.
