# Supplementary material for: The effects of diagnostic hysteroscopy on the reproductive outcomes of infertile women without intrauterine pathologies: a systematic review and meta-analysis
Source: Korean J Women Health Nurs. 2020 Dec 24;26(4):300–17. doi: 10.4069/kjwhn.2020.12.13 (PMC9328608; doi:10.4069/kjwhn.2020.12.13)
Supplement: Supplementary Table 1. — Characteristics of the included studies (N=11) [file kjwhn-2020-12-13-suppl1.pdf]

Supplementary Table 1. Characteristics of the included studies (N=11)

Study	Country	Study design	Group	n (PP population)	Age (year), mean ± SD	RIF history	Previous investigation	Method of pregnancy attempt
Tanakan et al. (2019) [36]	Turkey	Retrospective cohort study	OH<br>· Normal<br>· Abnormal<br>No OH	48<br>42<br>6<br>282	9.9 ± 4.3<br>-<br>-<br>30.3 ± 4.2	First	Normal HSG and TVS	IVF
Alleyassin et al. (2017) [35]	Iran	RCT	OH<br>· Normal<br>· Abnormal<br>No OH	110<br>85<br>25<br>110	29.55 ± 3.85<br>-<br>-<br>29.14 ± 4.34	First	Normal HSG, TVS, semen analysis, and hormonal profile	ICSI
El-Toukhy et al. (2016). [32]	European countries	RCT	OH<br>· Normal<br>· Abnormal<br>No OH	350 (323)<br>238<br>85<br>325 (348)	33.0<br>-<br>-<br>33.0	RIF	Normal TVS	IVF
Smit et al. (2016) [34]	Netherlands	RCT	OH<br>· Normal<br>· Abnormal<br>No OH	369 (325)<br>288<br>37<br>373 (364)	33 ± 4.4<br>-<br>-<br>33 ± 4.5	First	Normal TVS	IVF/ICSI
Pabuccu et al. (2016) [33]	Turkey	Retrospective cohort study	OH<br>· Normal<br>· Abnormal<br>No OH	119<br>58<br>61<br>244	30.7 ± 5.3<br>-<br>-<br>31.93 ± 4.4	RIF	Normal HSG and TVS	IVF/ICSI
Hosseini et al. (2014) [31]	Iran	Prospective cohort study	OH<br>· Normal<br>· Abnormal<br>No OH	142<br>103<br>39<br>211	32.6 ± 4.2<br>-<br>-<br>32.7 ± 4.3	RIF	Normal HSG and TVS	ART<br>IVF/ET
Kilic et al. (2013) [30]	Turkey	Prospective cohort study	OH<br>· Normal<br>· Abnormal<br>No OH	100<br>59<br>41<br>395	31.9 ± 3.4<br>-<br>-<br>31.4 ± 3.2	Uncertain	Normal HSG and TVS	IVF
Shawki et al. (2012) [29]	Egypt	RCT	OH<br>· Normal<br>· Abnormal<br>No OH	120 (105)<br>35<br>70<br>120	33 ± 11.14<br>-<br>-<br>31 ± 12.324	Uncertain	Normal HSG and TVS	ICSI
Makrakis et al. (2009) [28]	Greece	Prospective matched case-control study	OH<br>· Normal<br>· Abnormal<br>No OH	1,475<br>935<br>540<br>414	35.38 ± 3.96<br>35.8 ± 4.3-<br>36.2 ± 4.6<br>35.39 ± 3.95	RIF	Normal HSG	IVF/ICSI
Rama Raju et al. (2006) [27]	India	RCT	OH<br>· Normal<br>· Abnormal<br>No OH	255<br>160<br>95<br>265	-<br>27.40 ± 0.60<br>29.04 ± 0.92<br>26.72 ± 0.46	RIF	Normal HSG	IVF
Demirol and Gurgan (2004) [26]	Turkey	RCT	OH<br>· Normal<br>· Abnormal<br>No OH	210<br>154<br>56<br>211	-<br>35.4 ± 0.6<br>36.2 ± 0.1<br>34.3 ± 0.8	RIF	Normal HSG	IVF

ART: Artificial reproductive technology; ET: embryo transfer; HSG: hysterosalpingography; ICSI: intracytoplasmic sperm injection; IVF: in vitro fertilization; OH: office hysteroscopy; PP: per protocol; RIF: recurrent implantation failure; TVS: transvaginal sonography.

https://doi.org/10.4069/kjwhn.2020.12.13
