# Supplementary material for: The effects of diagnostic hysteroscopy on the reproductive outcomes of infertile women without intrauterine pathologies: a systematic review and meta-analysis
Source: Korean J Women Health Nurs. 2020 Dec 24;26(4):300–17. doi: 10.4069/kjwhn.2020.12.13 (PMC9328608; doi:10.4069/kjwhn.2020.12.13)
Supplement: Supplementary Figure 1. — Risk of bias. (A) Randomized controlled studies; summary. (B) Randomized controlled studies; by study. (C) Non-randomized studies; summary. (D) Non-randomized studies; by study. [file kjwhn-2020-12-13-suppl2.pdf]

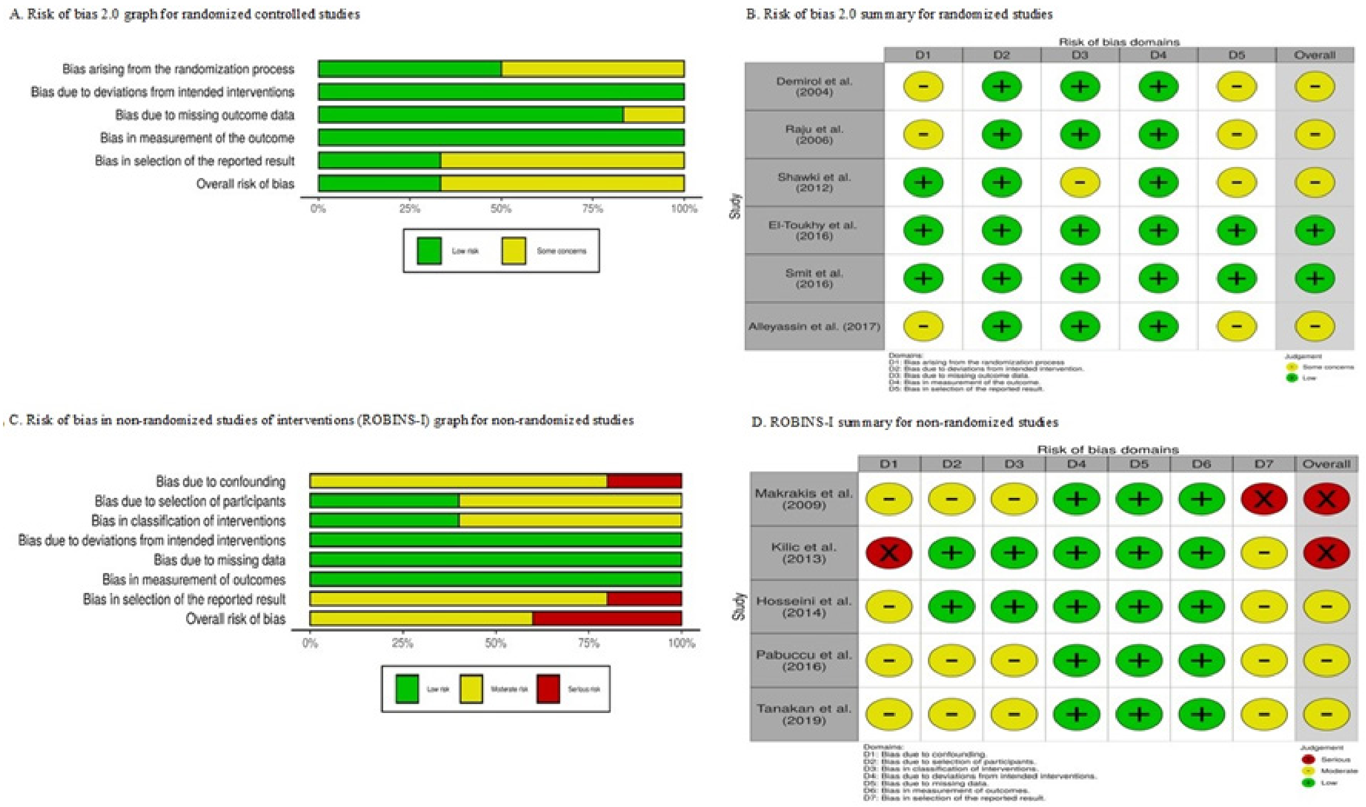

Supplementary Figure 1. Risk of bias. (A) Randomized controlled studies; summary. (B) Randomized controlled studies; by study. (C) Non-randomized studies; summary. (D) Non-randomized studies; by study.
